# LncRNAs as potential prognosis/diagnosis markers and factors driving drug resistance of osteosarcoma, a review

**DOI:** 10.3389/fendo.2024.1415722

**Published:** 2024-07-02

**Authors:** Siwang Hu, Xuebing Han, Gang Liu, Shuangshuang Wang

**Affiliations:** ^1^ The Orthopedic Center, Wenling First People’s Hospital (The Affiliated Wenling Hospital of Wenzhou Medical University), Wenling, China; ^2^ Hunan Provincial Engineering Research Center of Applied Microbial Resources Development for Livestock and Poultry, College of Bioscience and Biotechnology, Hunan Agricultural University, Changsha, China; ^3^ Department of Cardiology, Wenling First People’s Hospital (The Affiliated Wenling Hospital of Wenzhou Medical University), Wenling, China

**Keywords:** osteosarcoma, lncRNA, cancer cell, biomarkers, indicators

## Abstract

Osteosarcoma is a common malignancy that often occurs in children, teenagers and young adults. Although the treatment strategy has improved, the results are still poor for most patients with metastatic or recurrent osteosarcomas. Therefore, it is necessary to identify new and effective prognostic biomarkers and therapeutic targets for diseases. Human genomes contain lncRNAs, transcripts with limited or insufficient capacity to encode proteins. They have been implicated in tumorigenesis, particularly regarding the onset, advancement, resistance to treatment, recurrence and remote dissemination of malignancies. Aberrant lncRNA expression in osteosarcomas has been reported by numerous researchers; lncRNAs have the potential to exhibit either oncogenic or tumor-suppressing behaviors and thus, to govern the advancement of this skeletal cancer. They are suspected to influence osteosarcoma cell growth, replication, invasion, migration, remote dissemination and programmed cell death. Additionally, they have been recognized as clinical markers, and may participate in the development of multidrug resistance. Therefore, the study of lncRNAs in the growth, metastasis, treatment and prognosis of osteosarcoma is very important for the active prevention and treatment of osteosarcoma. Consequently, this work reviews the functions of lncRNAs.

## 1 Introduction to the treatment and research of osteosarcoma

**Figure 1:**
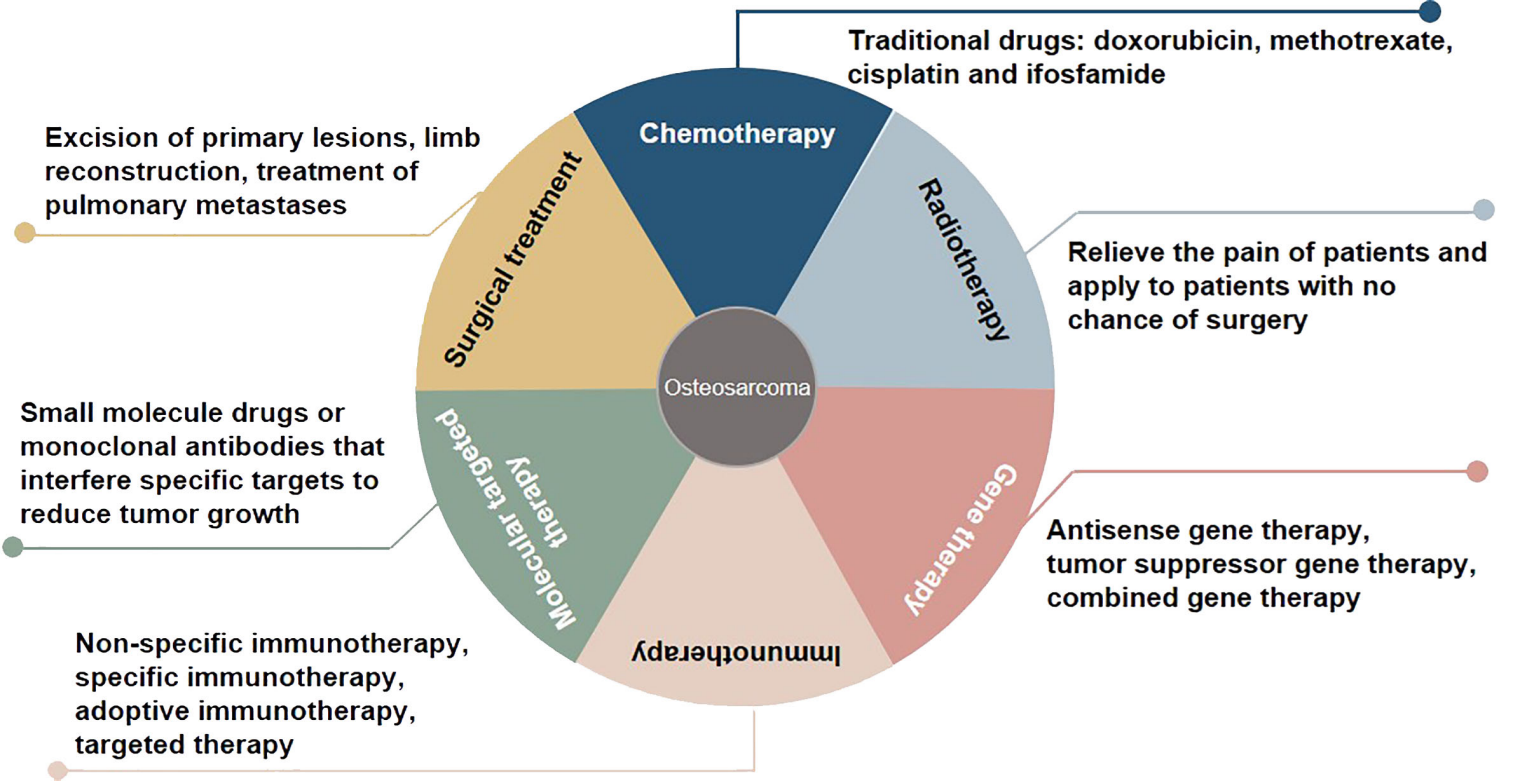
Overview of treatment methods for osteosarcoma.

Osteosarcoma, which originates from mesenchymal cells, is one of the most frequently occurring bone tumor. It is responsible for a significant proportion of child and teenage mortalities occurring due to malignancy. Typical histological appearances of osteosarcoma include spindle cells and the abnormal generation of osteoid (1). The long-term survival rate of individuals who present without remote tumor dissemination is approximately 80%. Conversely, when metastatic deposits are detected at diagnosis, the likelihood of long-term survival is lower than 20%, even when aggressive treatment regimens are instituted (2). At present, some progress has been made in the treatment of osteosarcoma, including chemotherapy, radiotherapy, gene therapy, immunotherapy, molecular targeted therapy, and surgical treatment (3, 4) (**Figure 1**). Due to the limitations of early medical development, the early surgical treatment of osteosarcoma is mainly based on amputations to save lives. However, the appearance and function of residual limbs after amputations adversely affect patients’ quality of life, causing long-term psychological distress. With the rapid development of medical science and technology, limb salvage treatment for osteosarcoma has replaced most amputations in clinical practice (5). Doxorubicin, methotrexate, cisplatin and, ifosfamide are known as classic drugs for osteosarcoma chemotherapy that have significantly improved the curative effect, survival rate, and quality of life, and reduced the harm to the human body caused by the side effects of treatment (6). The sensitivity of osteosarcoma to radiotherapy is relatively poor, and it is recommended to relieve the pain of patients and apply it to patients with no chance of surgery (7). Gene therapy, immunotherapy, and molecular targeted therapy are new methods for treating osteosarcoma, and their application prospects and values are incalculable. In the studies of whole genome sequencing and whole exome sequencing of osteosarcoma, some genes usually changed, including tumor suppressor genes *TP53*, *RB1*, *ATRX* and *DLG2* (8). Moreover, genomic instability of BRCA1/2-deficient tumors was observed in 80% of osteosarcomas (9). The combined genomic and transcriptome analysis of osteosarcoma samples also identified multiple fusion transcripts directly related to chromosome rearrangements. Although these fusion transcripts involve multiple loci in different cell lines and patient samples, the most common is affecting the *TP53* locus (10). Tailor-made therapies using biomarkers that predict responses to specific molecular targeted therapies are central concepts in precision medicine. Osteosarcoma mostly exhibits somatic changes in cell cycle and/or DNA damage repair pathways. Targeted therapy based on this abnormal prediction is a potential precise medical method. For example, abnormal expression of *TP53* impairs the G1 cell cycle checkpoint, thereby increasing the dependence of tumor cells on the G2 checkpoint to maintain DNA integrity and complete cell division (11). Therefore, drugs that disrupt the G2 checkpoint, such as WEE1 inhibitors, may enhance the activity of DNA damage agents and induce mitotic death in *TP53*-mutant osteosarcoma cells. However, most therapies are still in the stage of basic scientific research experiments, and only a few are used in clinical observation. In addition, the regulatory mechanism of osteosarcoma has yet to be clarified, so the therapeutic effect of targeted agents needs to be improved, which makes the use of contemporary treatments more challenging (12). Therefore, there is an urgent need to identify clinically relevant prognostic biomarkers for these patients.

Although several promising molecular markers have been detected in recent years, a diagnostic indicator particular to osteosarcoma has yet to be identified (13). Data are being accrued, which suggests that many malignancy-related genomic mutations occur in areas that, instead of encoding for proteins, frequently undergo transcription into IncRNAs. These are a cohort of non-protein transcripts of approximately 200 nucleotides in length (14).

LncRNAs contribute to the governance of gene expression via numerous mechanisms, for example, as epigenetic moderators, signals for the enhancement of transcription, transcription suppression decoys, or scaffolds for the generation of ribonucleoprotein complexes through engagement with proteins (15). The integrity and translation of mRNAs is influenced by lncRNAs; the latter also form antecedents to miRNAs and control their distribution. Overall, IncRNAs impact the expression of genes at both the transcriptional and post-transcriptional levels (15, 16). Additional pathways in which lncRNAs have been implicated encompass cell development, differentiation, and replication, together with the governance of the cell cycle and apoptosis (17, 18). They also play a significant part in the advancement and remote spread of a range of malignancies, for example, large intestine, hepatic, breast, bladder, and cervical neoplasias (19).

Even though research regarding the roles played by lncRNAs in osteosarcoma is still in its infancy, promising data imply that, in addition to their contribution to the control of numerous pathophysiological pathways, lncRNAs influence carcinogenesis as well as tumor advancement and remote dissemination (20, 21). They have also been utilized as biological markers, autonomous indicators of clinical prognosis and therapeutic targets, thus having notable diagnostic and therapeutic utility, as well as offering a method of tumor surveillance (22). Evidence suggests that the effect of chemotherapeutic drugs may be potentiated by lncRNAs, which influences patients’ prognosis (23). Therefore, this study reviewed the role of lncRNAs in osteosarcomas, which includes their influence on tumorigenesis, remote dissemination, clinical outcomes, and resistance to chemotherapeutic agents.

## 2 Classification and biological function of lncRNAs

**Figure 2:**
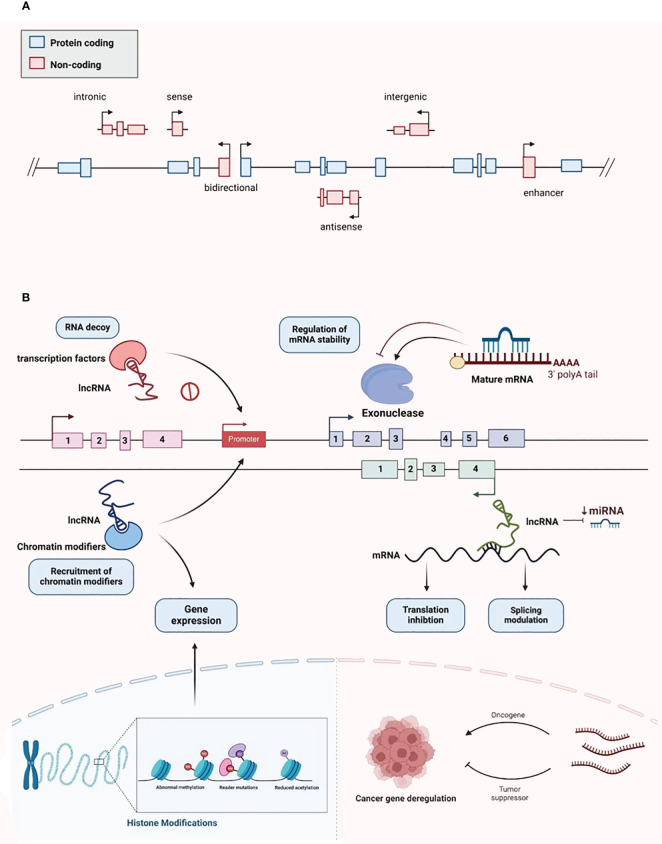
Classification and biological function of lncRNA. **(A)** LncRNA is divided into bidirectional, intergenic, intronic, antisense, sense, and enhancer lncRNA. **(B)** LncRNA plays various biological functions, including recruiting chromatin modifiers, regulating gene expression, participating in transcriptional silencing of protein coding genes, regulating mRNA stability, increasing mRNA expression, and being able to serve as key regulatory factors and oncogenes or tumor suppressors in tumor development and metastasis.

LncRNAs were first identified in murine transcripts (24). Typically, their length extend in excess of 200 nucleotides, and they have no capacity to encode proteins (24). They are categorized into the following types: bidirectional, intergenic, intronic, antisense, sense, and enhancer lncRNAs, depending on the configurational relationship between the lncRNAs and the genes for protein coding within the genome (**Figure 2A**) (25). RNA polymerase II is responsible for the transcription of most of these molecules, with a 5’cap and polyadenylated tail are common (26). Owing to the myriad transcripts, they were first deemed to be “noise” associated with the transcription process (27). However, more detailed contemporary work has demonstrated that lncRNAs have an abundance of physiological roles, such as the moderation of the epigenetic, transcriptional, and post-transcriptional expression of genes (**Figure 2B**). Additionally, they have considerable intracellular functionality, contributing to cell replication, differentiation, cell cycle advancement, growth, and programmed cell death (28, 29). The alteration and remodeling of chromatin and histone, together with the localization of the nuclear body, are mechanisms via which gene expression can be modified by lncRNAs (30). Chromosomal configurations can also be adjusted by lncRNAs in concert with the SWI/SNF complex, a process that immediately impacts gene expression (31). Although lncRNAs are not attributed to coding protein, several are suspected of involvement in silencing protein encoding genes during transcription through modification of the structure of chromatin and the conscription of complexes that impact this process (32).

In stem cells, the differentiation of cells is influenced by lncRNAs; in somatic and pluripotent stem cells, sizeable intergenic non-coding RNAs can promote cellular reprogramming (33). Furthermore, in addition to immediately influencing target expression, lncRNAs also have the ability to govern expression through secondary pathways, for example, acting as ‘molecular sinks” for signaling molecules, such as RNA-binding proteins that moderate chromatin (32). A key mechanism for their ability to control gene expression is that they act as competing endogenous RNAs (ceRNAs) for miRNAs, reducing the bioavailability of miRNAs and enhancing target mRNA titers (34). Several studies have additionally demonstrated that lncRNAs work as key controlling molecules that behave as oncogenes or tumor-inhibiting agents. These are essential actors in numerous forms of malignancy, with the potential to promote the progression of carcinogenesis, remote tumor dissemination, and resistance to chemotherapeutic agents (35–38).

## 3 LncRNAs in osteosarcoma

### 3.1 Up-regulated lncRNAs in osteosarcoma

The potential oncogenic effects of lncRNAs in osteosarcoma described in previous studies are presented in **Table 1**. When samples from osteosarcomas were compared with neighboring normal tissues, the expression of the lncRNA TUG1, which is situated on chromosome 22q12.2 and is 7.1 kb in size, was noted to be amplified (2). This finding was related to adverse prognosis, forming an autonomous predictive indicator of overall survival (39). *In vitro* studies have demonstrated that cell replication is inhibited in the presence of TUG1 knockdown, which is associated with the arrest of the G0/G1 cell cycle and programmed cell death. Additionally, *in vivo* work has shown that TUG1 knockdown leads to a reduction in the growth of cancerous lesions (39). TUG1 can promote the proliferation of cancer cells. When TUG1 is inhibited, the proliferation of cancer cells decreases, and the replication efficiency of osteosarcoma cells decreases. Recent study has also found that TUG1 can act as a miR-26a-5p sponge and promote the progression of osteosarcoma by up-regulating ZBTB7C (40), so TUG1 can be used as a diagnostic marker for osteosarcoma, targeting TUG1 may be an effective strategy for the treatment of osteosarcoma.

**Table 1:** Roles and function mechanisms of lncRNAs in osteosarcoma.

LncRNA	Expression	Function	First recognized	Role and mechanism
TUG1	Up	Oncogene	*in vivo*	Inhibits G0/G1 cell cycle arrest and apoptosis, and promotes cell proliferation by sponging miR-9-5p and miR-26a-5p
MALAT1	Up	Oncogene	*in vivo*	Promotes cell proliferation and metastasis by activating PI3K/AKT signaling pathway
H19	Up	Oncogene	*in vivo*	Induced by Hh signal transduction and Yap1 overexpression, thereby promoting the development of osteosarcoma
NEAT1	Up	Oncogene	*in vitro*	Enhances malignant features of osteosarcoma cells through sponging miR-579, and increase the EMT of osteosarcoma cells through sponging miR-438
DANCR	Up	Oncogene	*in vitro*	Enhances proliferation, migratory potential, invasiveness and autophagy of osteosarcoma cells by sponging miR-216a-5p
SPRY4- IT1	Up	Oncogene	*in vitro*	Enhances the osteosarcoma cell growth, migration and invasion through the inhibition of G1 arrest; Regulates the expression of ZEB1 and ZEB2 by sponge miR-101
LSINCT5	Up	Oncogene	*in vivo*	Plays a carcinogenic role by inhibiting the expression of adenomatosis polyposis coli; Promotes the proliferation, migration, and invasion of osteosarcoma cells
BCAR4	Up	Oncogene	*in vitro*	Promotes the progression of osteosarcoma by activating the GLI2 signaling pathway
UCA1	Up	Oncogene	*in vivo*	Enhances cell cycle progression through different mechanisms; interacts with various cancer-related signaling pathways such as mTOR, AKT, Wnt, Hippo and JNK pathways
ANRIL	Up	Oncogene	*in vivo*	Increases cell proliferation
Loc285194	Down	Tumor suppressor	*in vivo*	Inhibits the growth of tumor cells through inhibiting miR-211 expression
MEG3	Down	Tumor suppressor	*in vitro*	Reduces overall survival by regulating the expression of p53; Promotes osteosarcoma chemosensitivity by regulating anti-tumor immunity through the miR-21-5p/p53 pathway and autophagy
FER1L4	Down	Tumor suppressor	*in vivo*	Regulates EMT and cell apoptosis by serving as a sponge for miRNA-18a-5p
GAS5	Down	Tumor suppressor	*in vivo*	Suppresses osteosarcoma cell growth and invasion by affecting the PI3K/AKT pathway; Enhances the sensitivity of osteosarcoma cells to Cisplatin through the GAS5/miR-26b-5p/TP53INP1 axis
TRP53COR1	Down	Tumor suppressor	*in vivo*	Suppresses the proliferation of osteosarcoma cells through sponging miR-130b
SRA1	Down	Tumor suppressor	*in vitro*	Reduces cell proliferation, invasion, and migration via sponging miRNA-208a

Another lncRNA that exhibits excessive expression in osteosarcoma is metastasis-associated lung adenocarcinoma transcript 1 (MALAT1), which is also termed nuclear-enriched transcript 2 (NEAT2) and mascRNA. It comprises 8700 nucleotides and is present on a locus of chromosome 11q13 (41–43). MALAT1 is not polyadenylated but instead contains a highly unusual 3’-terminal structural motif designated as the stability element for nuclear expression (ENE), which is essential for the stability of mature transcripts and the subsequent accumulation and carcinogenic activity of MALAT1. Therefore, cells are free from the influence of cellular degradation pathways, leading to MALAT1’s sustained carcinogenic activity in a variety of cancer types (44, 45). It was originally identified as a biological indicator of metastatic disease in the initial phases of non-small cell lung cancer (46). In osteosarcoma samples, MALAT1 expression was increased, a finding that has a positive association with metastatic deposits in the lungs (47). Furthermore, the expression of phosphorylated PI3Kp85α, matrix metallopeptidase 9 (MMP-9), proliferating cell nuclear antigen (PCNA), and Akt were significantly inhibited in MALAT1-deficient cells. The replication and migration of osteosarcoma cells was inhibited following MALAT1 knockdown. Additionally, in MNNG/HOS and U2OS cells, the generation of tubular network configurations was suppressed, and stress fibers were fractured (48). The up-regulated expression of MALAT1 is closely related to the presence of distal osteosarcoma deposits, and the inhibition of MALAT1 can reduce the migration and invasion of osteosarcoma cells. In addition, it has been shown that, METTL3 in osteosarcoma cells promotes the m6A modification of MALAT1, and enhances the carcinogenic function of MALAT1 (49). One of the key oncogenic pathways in osteosarcomas affecting humans is the phosphatidylinositol 3-kinase (PI3K)/Akt pathway (50, 51). It has several major functions relating to cancer cell growth, apoptosis, and migratory and invasive properties (52). Diminishing MALAT1 expression led to lower levels of phosphorylated PI3K p85α and Akt (47). MALAT1 exhibits competitive bonding to miRNA-129-5p and therefore prevents this molecule from promoting the breakdown of RET. Subsequently, the rise in RET concentrations enhances the activity of the PI3K/Akt signaling pathway (53). Additionally, MALAT1 demonstrated oncogenic functions, increasing osteosarcoma cell growth potentially through the stimulation of the PI3K/AKT and RhoA/ROCK pathway (47, 48).

Initially described as a molecule that could inhibit epithelial cell differentiation, differentiation antagonizing non-protein coding RNA (DANCR or ANCR), which has its locus on human chromosome 4q12, has also been shown to enhance the stemness properties of cell lines from hepatocellular carcinoma (54, 55). DANCR can promote the pathogenesis of osteosarcoma, and the silencing of DANCR has been demonstrated to promote programmed cell death, using functional assays, to inhibit the replication, migratory ability, invasiveness, and autophagic capability of cells from osteosarcomas. The expression of SOX5 and levels of the receptor tyrosine kinase AXL are amplified by DANCR owing to its sponge-like function in relation to miR-216a-5p and miR-33a-5p (56), respectively. Moreover, METTL3 promotes osteosarcoma progression by increasing the stability of DANCR mRNA through m6A modification, which means that METTL3 may be a promising therapeutic target for osteosarcoma treatment (57).

Another lncRNA, H19, is 2.3 kb in size and is near the telomeric region of human chromosome 11p15.5. It is implicated in the governance of the expression of IGF2 (58). In osteosarcomas, an abnormal degree of H19 expression has been detected, which can be promoted by amplified Hedgehog (Hh) signaling and upregulated yes-associated protein 1 (Yap1) (59). This observation substantiates the theory that erroneous Hh signaling expression in osteoblasts plays a key role in the osteosarcoma associated with the overexpression of H19 and Yap1. Gallic acid inhibits tumor growth in osteosarcoma cells through the H19-mediated Wnt/β-catenin signaling regulatory axis (60).

The familial tumor syndrome multiple endocrine neoplasia type 1 locus, situated on chromosome 11, gives rise to the transcription of the lncRNA NEAT1 (61). Its oncogenic properties have been demonstrated to augment the neoplastic characteristics of cells from osteosarcomas (62). By sponging miR-579 and upregulating MMP13, the role of NEAT1 in the epithelial-mesenchymal transition (EMT) is promoted (63). NEAT1 can also sponge miR-438, increase the expression of STAT3, and inhibit that of STAT1, and then increase the EMT of osteosarcoma cells (64).

Another lncRNA, SPRY4-IT1, which has a size of 708 bp, originates from a SPRY4 intron on chromosome 5q31.3, which contains the coding for an intrinsic receptor-transduced mitogen-activated protein kinase pathway inhibitor (65, 66). The expression of this lncRNA was observed to be amplified in cell lines and specimens from osteosarcomas, while the knockdown of it inhibited the invasive, migratory, and replicative properties of the malignant cells through the generation of G1 cell cycle arrest and heightened apoptosis (67). SPRY4-IT1 can also regulate the expression of ZEB1 and ZEB2 by sponge miR-101 activity, thereby promoting the progression of osteosarcoma (68).

Compared with adjacent normal tissues, long stress-induced non-coding transcript 5 (LSINCT5) was significantly up-regulated in osteosarcoma tissues. The abnormal expression of LSINCT5 is usually associated with cancer progression and poor prognosis, and high LSINCT5 expression is associated with advanced Enneking stage, large tumor volume, high histological grade, and current distant metastasis (69). The overexpression of LSINCT5 can promote the proliferation, migration, and invasion of osteosarcoma cells *in vitro*, while the inhibition of its expression has the opposite effect (69). The exploration of the mechanism displayed that LSINCT5 can interact with EZH2 to inhibit the expression of adenomatosis polyposis coli, a negative regulator of the Wnt/β-catenin pathway, to play a carcinogenic role (70). In general, LSINCT5 plays a carcinogenic role in osteosarcoma cells and may be a predictor of the clinical outcomes of osteosarcoma patients, and a promising candidate for osteosarcoma prognosis and treatment.

In addition, there are some other common lncRNAs that are up-regulated in osteosarcoma. For example, the expression of BCAR4 (71), HULC (72), UCA1 (73), and ANRIL (74) in osteosarcoma tissues is significantly higher than that in osteoblasts and adjacent tissues. After knocking down BCAR4, the proliferation, invasion and migration of osteosarcoma cells were inhibited. In addition, BCAR4 promotes the progression of osteosarcoma by activating the GLI2 signaling pathway (75). UCA1 can act as a sponge for many tumor suppressor miRNAs (73), enhance cell cycle progression through different mechanisms (76), and interact with various cancer-related signaling pathways such as mTOR, AKT, Wnt, Hippo and JNK pathways (77). Overexpression of ANRIL increases cell proliferation, and higher ANRIL expression is significantly associated with death and metastasis (78).

### 3.2 Down-regulated lncRNAs in osteosarcoma

**Table 1** illustrates several cancer-suppressing lncRNAs that exhibit diminished expression in osteosarcoma.

The lncRNA, Loc285194, which is also named the LSAMP antisense RNA 3, has its locus on chromosome 3q13.3. It comprises 4 exons and is 2105 nucleotides long (79). Its tumor-suppressing activity is moderated via p53 due to the inhibition of the expression of miR-211 (80). In specimens and cell lines from primary osteosarcomas, a lack of Loc285194 expression has been demonstrated. When Loc285194 was depleted, the replication of normal osteoblasts was enhanced because of its effects on the cell cycle, apoptotic transcripts, and the expression of VEGF receptor 1 (79).

MEG3, is a member of the DLK1–MEG3 locus found on human chromosome 14q32.3 (81). Its functions include the accrual of protein p53, the transcription of p53-dependent promoters, and the exclusive moderation of the expression of p53 target genes (82, 83). When contrasted with neighboring benign tissue samples, there was an obviously reduced MEG3 expression in tissues from osteosarcomas. This observation was correlated with the disease stage and the presence of remote metastases (*P* < 0.05) (2). This gene is associated with the Notch, and TGF-β pathways (84); the amplified expression of MEG3 led to a suppression of TGF-β, Notch1, N-cadherin, and Hes1 expression, and the upregulation of the expression of E-cadherin (85). Mechanistically, MEG3 promotes osteosarcoma chemosensitivity by regulating anti-tumor immunity through the miR-21-5p/p53 pathway and autophagy, demonstrating that MEG3 may be a promising therapeutic target for osteosarcoma chemoresistance (86).

A range of malignant pathologies is influenced by the biological activities of FER-1 family member 4 (FER1L4), a lncRNA that has a size of 6.7 kb and is situated on chromosome 20q11.22 (87). Compared to normal tissues, titers of this molecule were notably reduced in samples and cell lines from osteosarcomas. Additionally, the osteosarcoma-enhancing miRNA, miRNA-18a-5p, which has been proposed to be the target molecule for FER1L4 in this malignancy, was recognized regarding the governance of programmed cell death and EMT (52).

Moreover, chromosome 1q25 is the location of the *de novo* tumor-suppressor, growth arrest-specific transcript 5 (GAS5). This lncRNA is a 5’-terminal oligopyrimidine RNA that comprises a dozen non-conserved exons and approximately 630 nucleotides (88). Its expression is diminished in breast, stomach, lung, and prostate tumors, as well as in additional neoplasias (89, 90). Its expression is also downregulated in osteosarcoma samples and cell lines in humans when judged against neighboring areas and normal osteoblast cells. Functionally, it acts as a ceRNA, sponging miR-23a-3p to amplify the expression of PTEN and inhibit osteosarcoma cell replication and invasion through its effect on the PI3K/AKT pathway (91). In addition, GAS5 also enhances the sensitivity of osteosarcoma cells to Cisplatin through the GAS5/miR-26b-5p/TP53INP1 axis, which may be a potential indicator for the treatment of osteosarcoma (92).

In addition, the replication of osteosarcoma cells is subject to modification by lncRNA-p21, which is also referred to as TRP53COR1. This is associated with amplified PTEN expression due to the replication of osteosarcoma cells being subject to modification by lncRNA-p21, which is also termed TRP53COR1. This is associated with amplified PTEN expression because of sponging miR-130b, an oncomiR (64). As a principal gene dictating cancer suppression in humans, PTEN is responsible for the dephosphorylation of AKT at Thr 308 and Ser 473, which leads to the inhibition of the signaling potency of AKT and, consequently, a reduction in cell growth and the enhancement of apoptosis (65).

A further lncRNA, which operates as a coactivator of RNA, SRA1 has been proposed to be a major actor in myogenesis, steroidogenesis, breast carcinogenesis, and cardiac muscle disorders. Anomalous levels of expression of SRA1 have been identified in several malignancies in humans (93). An anti-cancer function of SRA1 was observed in osteosarcomas, in which SRA1 diminished the migratory, replicative, and invasive properties of the tumor cells and also acted as a sponge for miRNA-208a, thus promoting apoptosis (94).

## 4 LncRNAs regulate the cell proliferation, invasion and metastasis of osteosarcomas

**Figure 3:**
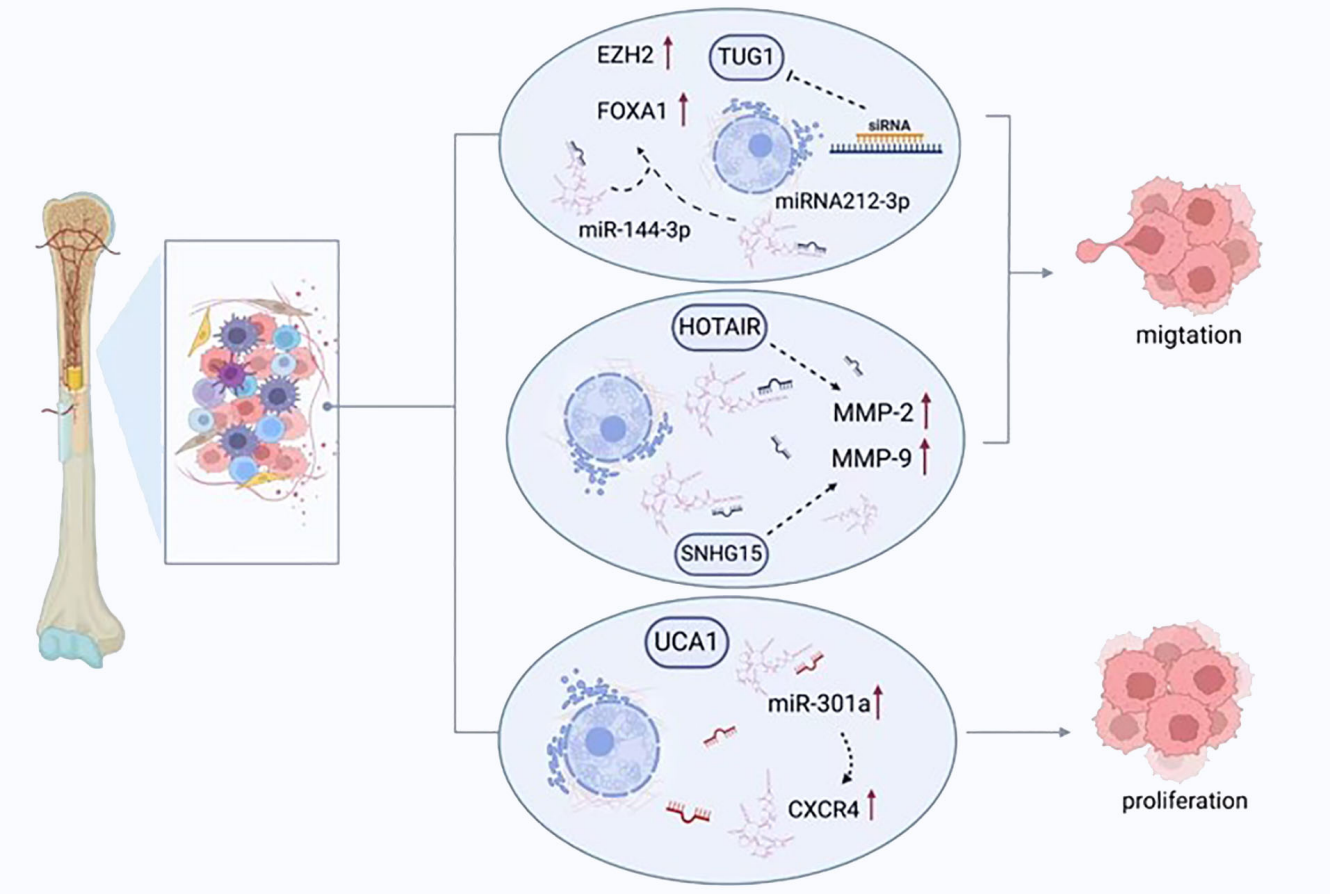
LncRNAs regulate the migration, proliferation, and apoptosis of osteosarcoma cells. TUG1 promotes the expression of FOXA1 and enhancer of zeste homolog 2 (EZH2) by competitively binding to miRNA-212-3p and miR-144-3p, thereby enhancing the proliferation of cancer cells. UCA1 enhances the migration of tumor cells by increasing the expression of miR-301a and chemokine receptor-C-X-C motif chemokine receptor 4 (CXCR4). HOTAIR and SNHG15 promote cell proliferation by regulating the expression of MMP-2/MMP-9.

Ongoing cell growth and replication without regulation is a pathognomonic sign of tumorigenesis; promoting the removal of malignant cells is deemed the definitive goal of therapy (95). Carcinogenesis can be facilitated by activating oncogenes and inhibiting tumor suppressor genes (96). Various lncRNAs display these functions in osteosarcomas, modifying either the cell cycle or apoptosis, and regulating cellular replication or migration (**Figure 3**).

### 4.1 LncRNAs regulate the cell proliferation and migration of osteosarcomas

In samples from osteosarcoma, a polymerase chain reaction was utilized to quantify the levels of hypoxia-inducible factor-2α (HIF2α) promoter upstream transcript (HIF2PUT). The results indicated that HIF2PUT inhibited tumor stem cells through its effects on the expression of HIF2α. Significant inhibitory influences were observed on cellular replication and migration in the presence of amplified HIF2PUT expression. The proportion of CD133-expressing cells was reduced and the MG63 cells evidenced a diminished capacity to form osteosarcoma stem spheres (19, 97). Conversely, when HIF2PUT was depleted via siRNA, there was a marked enhancement of cell growth and migration (97).

It has also been determined that replication and colony generation in cells from osteosarcomas can be suppressed by tumor suppressor candidate 7 (TUSC7) (43). Its knockdown with siRNA caused increased cellular replication and the presence of cell colonies, together with a decrease in programmed cell death. However, there was no impact on the cell cycle (98). The amplification of the expression of the angiomotin (AMOT) gene in cells from human osteosarcomas by SNHG12 also has been reported to accelerate cellular replication and migration, while the knockdown of SNHG12 had the opposite consequence but failed to influence programmed cell death (99). When SNGH12 and AMOT underwent knockdown, attenuated cell migration and growth were detected (99). Notch signaling is a pathway that is extremely conserved, underpins many biological activities, and contributes to the pathogenesis of numerous malignancies (100). Notch signaling is responsible for the governance of a number of lncRNAs and is regulated by them simultaneously. SNHG12 stimulates the Notch signaling pathway and precipitates enhanced oncogenesis and osteosarcoma dissemination by sponging miR-195-5p (101). Research has observed that the growth and replication of osteosarcoma cells was impeded by atypical lncRNA expression. A lack of TUG1 diminishes the proliferation of cancerous cells and pauses the cell cycle at the stage, G0/G1 (102), additionally, it also acts as a ceRNA to sponging miRNA-212-3p and miR-144-3p (103). Subsequently, the FOXA1 and enhancer of zeste homolog 2 (EZH2) expression was increased, which are target genes for the transcription of oncogenes (103). The potency of osteosarcoma cell replication was diminished, and the apoptosis was enhanced with the suppression of TUG1 with siRNA (104). The arrest of the cell cycle and apoptosis are both triggered following MALAT1 knockdown, which also leads to the suppression of the growth of osteosarcoma and its dissemination (48).

The continuation of an undifferentiated cell type is influenced by a *de novo* recognized oncogenic lncRNA, DANCR (19). In U2OS and SAOS cells, the replication of the cells was markedly reduced by DANCR knockdown, which also reduced colony generation within U20S cells and paused the cell cycle in the latter at stage, G0/G1. The endogenous levels of proteins associated with the cell cycle, such as p21, CDK2, and CDK4, are governed by DANCR (105). miRNA-335-5p and miRNA-1972 expression are also promoted by DANCR, which accelerates replication that is induced via ROCK1 and ceRNA network transfer and consequently promotes the pathogenesis of osteosarcomas (106).

The amplified expression of TMPO antisense RNA 1 (TMPO-AS1) has been observed in osteosarcomas. Conversely, samples and cell lines from osteosarcomas have evidenced the downregulation of miR-199a-5p (107). The inhibition of cellular replication and enhanced programmed cell death were identified following TMPO-AS1 knockdown, and the regulation of WNT7B occurred via the immediate sponging of miR-199a-5p, which suppressed Wnt/β-catenin activity (108). Furthermore, the WNT7B knockdown salvaged the miR-199-5p inhibitor’s suppressive effect on osteosarcomas, which could be eradicated by administering the Wnt pathway stimulator lithium chloride.

In individuals with osteosarcoma, a poor prognosis was suggested by the amplified expression of bladder cancer-associated transcript 1 (BLACAT1) (109). This was linked with promoted cellular replication and invasive properties, whereas a reduction in BLACAT1 expression was associated with the opposite effect. Interestingly, the enhancement of tumor cell replication and migratory function was achieved through an effect on STAT3 phosphorylation.

Another major influence on the onset and progression of osteosarcoma is the modified frailty index 2 (MFI2), which MMFI2 promotes the proliferation and migration of osteosarcoma cells by regulating the expression of forkhead box P4 (FOXP4) (110). Cellular replication within osteosarcomas has been demonstrated to be enhanced by P50-associated COX-2 extragenic RNA (PACER). The methylation of DNA has a regulatory influence on PACER, which arises through the NF-κB-dependent stimulation of the gene, COX-2 (111). Triggering ZEB1 transcription via ZEB1 Antisense 1 (ZEB1-AS1), which has oncogenic characteristics, also induces the replication of osteosarcoma cells (112). In cells from human osteosarcomas, oncogenic lncRNA SNHG12 additionally impacts cellular replication through angiomotin gene expression amplification (99). Osteosarcoma cell replication was suppressed by reduced HNF1A-antisense 1 (HNF1A-AS1) expression, an action mediated through Wnt/β-catenin cascade inactivation (113).

### 4.2 LncRNAs regulate the cell apoptosis of osteosarcomas

The interaction between EZHW and HOXD-AS1 reduces the expression of p57 and promotes the development of osteosarcoma (114). The most common arising Apo-ERα-regulated lncRNA (AER-lncRNA) in mammary tumors is down syndrome cell adhesion molecule anti-sense RNA 1 (DSCAM-AS1) (115), which has been found to have a high level of expression within cell lines from osteosarcomas. Apoptosis in osteosarcoma has been markedly accelerated through the DSCAM-AS1 knockdown and inhibition of the Wnt pathway (116).

### 4.3 LncRNAs regulate the invasion and metastasis of osteosarcoma

The main clinical issues relating to osteosarcomas are remote tumor dissemination and the likelihood of recurrence, which hinders the efficacy of therapies and leads to poor prognosis in individuals with this type of malignancy. Cancerous deposits distant to the primary lesion occur over several pathological stages via a complicated mechanism that comprises regional tumor invasion, intravasation, remote spread, extravasation, and colonization (22). At the original tumor site, the interplay between cells and the extracellular matrix (ECM) is changed by malignant cells. These cells break away from the primary location and extend into neighboring structures, and gain passage through the circulation to remote viscera, where they attach to the vasculature walls and leak into their target tissues before replicating from microscopic colonies to generate secondary neoplastic deposits (98). Genetic and epigenetic abnormalities usually underlie this clinical process (117, 118). Metastatic tumors in osteosarcomas frequently occur in the lungs; these are challenging to address and give rise to the most common mode of death, respiratory failure (119). It is thought that about 85% of individuals with skeletal malignancy present with remote tumor deposits (120).

A group of proteolytic enzymes, MMPs, play a key role in the invasion and dissemination of cancerous cells through their capacity to breakdown the ECM and basement membrane, thus remodeling the microenvironment of the primary lesion and encouraging the formation of a malignant neovasculature. In many human malignancies, increased levels of MMP-2 and MMP-9 are released, an observation linked with an adverse clinical prognosis (121). Amplified HOTAIR expression accelerates osteosarcoma cell invasion through the elevated liberation of MMP-2 and MMP-9 (122). Conversely, reduced HOTAIR expression in cell lines from osteosarcomas, including U2OS, 143B, MNNG/HOS, and MG-63, suppresses malignant cell replication and invasion as well as inhibits the release of MMP-2 and MMP-9 (122). The growth of osteosarcoma can be inhibited in U20S cells with HOTAIR knockdown following implantation in xenograft models (122). SNHG15 can also promotes cell proliferation and invasion by regulating the expression of MMP-2/MMP-9. In addition, MMP-2 and MMP-9 expression can be enhanced by LINC00968 amplification, leading to ECM breakdown and encouraging migration and invasion by cancerous cells (123).

The evidence that lncRNAs are linked with signaling pathways, which are key actors in controlling osteosarcoma cell invasion, migration, and dissemination, is irrefutable. LncRNA plays a role in the development of osteosarcomas by affecting the wnt pathway. For example, when cell lines from osteosarcomas are contrasted with benign osteoblastic cells, the expression of the lncRNA gastric carcinoma proliferation enhancing transcript 1 (GHET1) is amplified. Knockdown of this molecule has been demonstrated to suppress the migration of osteosarcoma cells, together with their invasion and EMT. To some extent, these events are mediated through the control of the Wnt/β-catenin pathway. Compared with the control group, the expression levels of Wnt and β-catenin were decreased after GHET1 knockdown (124). Elevated levels of the lncRNA, CRNDE, have been identified in cell lines and tissue samples from osteosarcomas, with the knockdown of CRNDE leading to limited cellular invasion, the reduced expression of N-cadherin, vimentin, and snail, and amplified E-cadherin and ZO-1 expression (125). A potential mechanism for this process may be the activation of the Wnt/β-catenin signaling pathway via the increased phosphorylation of GSK-3β (125). The upregulation of MALAT1 expression strongly relates to the presence of remote osteosarcoma deposits. This lncRNA can bind to miR-144-3p and enhance ROCK1/2 levels which contribute significantly to the metastatic process (126). MALAT1 may inhibit tumor growth and metastasis through PI3K/AKT signaling pathway (47). The *in vivo* and *in vitro* lentivirus-mediated siRNA reduction of MALAT1 expression decreases in PCNA, MMP-9, p-PI3K, and RhoA/ROCKs expression, which then attenuates the growth, invasion, and dissemination of osteosarcoma cells (47, 48). MiR-184, as well as β-catenin, TCF4, and c-MYC, which are downstream Wnt signaling pathway factors, is negatively impacted by MEG3, which inhibits the replication and the migration of osteosarcoma cells *in vitro* and cancerous growth *in vivo* (127). The transition of epithelial cells into a mesenchymal phenotype is known as enforced EMT, which enhances tumor invasiveness and, clinically, leads to worse overall survival (128). This process can be recognized by a rise in mesenchymal indicators, including N-cadherin, Slug, Twist, Vimentin, and Fibronectin, and by a reduction in epithelial indicators such as E-cadherin (12). The Hh signaling exhibits anomalous activity in cell lines from osteosarcomas and samples from primary human osteosarcomas, which promotes migration and the onset of osteoblastic osteosarcoma (129). In mice with a heterozygous p53 background with amplified Hh pathway stimulation, skeletal cancers occurred freely, even at 7 months of age, in association with marked rises in the potent oncogenes Yap1 and H19 (59). Notably, cancer progression can be suppressed by silencing either of these genes (130).

Tumor cell migration and invasion can also be influenced by lncRNAs via miRNA. Cell survival, migration, and invasion were improved by urothelial carcinoma associated 1 (UCA1) (131), with the expression of this molecule having a positive correlation with chemokine receptor-C-X-C motif chemokine receptor 4 (CXCR4) and miR-301a. Additionally, the expression of miR-301a was amplified, which consequently heightened the expression of CXCR4 (107). Some studies have reported a robust link between the degree of CXCR4 expression and the invasive and metastatic properties of osteosarcoma (132, 133). Moreover, miR-301a has been demonstrated to display malignant functionality in osteosarcomas and additional human malignancies (134). Furthermore, amplified miR-301a expression can prevent the suppressive action of knockdown of UCA1 in cells from osteosarcomas, a process that can be reversed by inhibiting CXCR4. ATB has demonstrated an increase in cancer. In individuals with osteosarcomas, such raised levels lead to greater ZEB1 and SEB2 expression through the suppression of miRNA-200. These findings are associated with a late Enneking stage, remote tumor dissemination, and adverse survival statistics (135). When ATB is lacking, the use of shRNA has a marked inhibitory impact on the growth, migration, and invasion of osteosarcoma cells.

Augmented cancer dimensions, late Enneking stage, remote dissemination, and adverse survival rates were linked with FGFR3 antisense transcript 1 (FGFR3-AS1), which enhanced the stability of FGFR3 mRNA and amplified the expression of FGFR3 by coupling the antisense with FGFR3 3′-UTR (19). When FGFR3-AS1 underwent knockdown, the *in vitro* growth of osteosarcoma cells within a xenograft was inhibited, and similar findings were seen *in vivo* (136).

In contrast to the oncogenic function of many lncRNAs, some operate in a tumor-suppressive capacity. The depletion of NBAT1 accelerates tumor cell replication and invasion (137), while excessive NBAT1 expression suppresses these events and tumor cell migration by inhibiting the effects of miR-21 and impacting its targeted gene (138). Influencing the miR-34a-5p/Sirt1 network via the knockdown of CAMK2D-associated transcript 1 (C2dat1) has also been demonstrated to diminish the invasive and migratory abilities of osteosarcoma cells (139).

## 5 LncRNAs’ regulation of other signaling pathways in osteosarcoma

**Figure 4:**
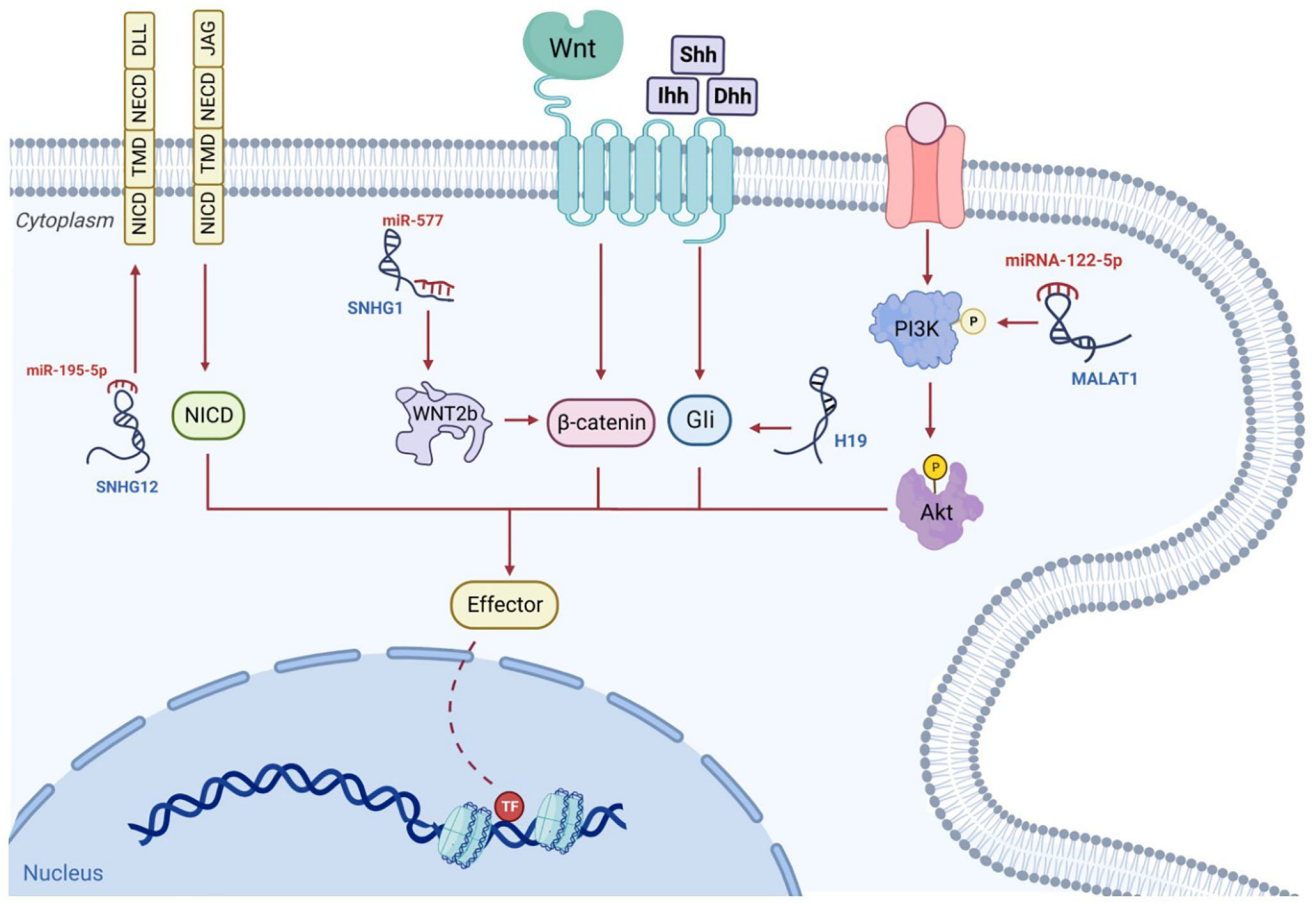
The signaling pathways associated with osteosarcoma are governed by lncRNAs. For example, the lncRNA, SNHG, behaves as a competitive miRNA blocker and increases the stability of β-catenin; subsequently, the triggered β-catenin is transported to the nucleus, where it instigates the transcriptive process. The competitive binding of MALAT1 to miRNA-122-5p can occur. This enhances phosphorylated PI3K and AKT levels which influence osteosarcoma progression. The hedgehog ligands, Shh, Ihh and Dhh, enhance Gli segregation from the microtube, after which Gli enters the nucleus to commence transcription. This mechanism may be enhanced by additional lncRNAs such as H19. NICD, TMD and NECD make up the lone transmembrane protein known as the Notch receptor. Ligands, including DLLs and JAGs, which are situated on adjacent cell walls, attach to their extracellular component, NECD, and switch on the Notch signaling cascade. The stimulated NICD is liberated into the cytoplasm to trigger transcription. Additional lncRNAs such as SNHG12, can increase Notch2 expression and facilitate this mechanism.

As a major contributor to cellular pathways, cell signaling has a significant influence on the modification of the expression of numerous genes in a spectrum of malignancies. A recent study has indicated that the control of many signaling cascades in osteosarcoma is impacted by lncRNAs, which results in changes to cell replication, differentiation, and programmed cell death (**Figure 4**). In advancing malignant disease in humans, Wnt signaling is a notable and well-recognized example (140). Changes in the constituents of this pathway, such as mutations, amplifications, deletions, hypermethylation of promoters, and changes in localization within the subcellular level, are considered some of the most significant mechanisms involved in the onset and development of osteosarcomas (141). In this malignancy, the regulation of several components, such as ligands, receptors, co-receptors, and antagonists, is disrupted, this finding suggests that this cascade plays a major role in promoting malignant characteristics and tumor metastasis (142). In individuals with osteosarcomas, there is marked enhancement of the activity of the Wnt signaling cascade, and the accrual of the downstream transcription factor, β-catenin, in tissue specimens (143). It has been suggested that this factor could be stabilized with the competitive blockade of miRNA in relation to mRNA binding, and through the conscription of RNA-binding protein to mRNA, processes facilitated by lncRNAs (144–146). There is further potential for lncRNAs to form a complex with DNA and to modify gene expression, such as amplifying the transcription of β-catenin and thus downregulating the expression of the Wnt pathway inhibitor (70). The amplified expression of the lncRNA, BE503655, has been identified in cell lines and samples from osteosarcomas (15), with the mRNA levels and protein expression of β-catenin markedly diminished in this tumor following BE503655 knockdown by regulating Wnt/β-catenin pathways (145). Equivalent findings are present for the lncRNA CAT 104, and the small nucleolar RNA host gene 1 (SNHG1), with a significant rise in their expression. They influenced the Wnt cascade by operating as ceRNAs sponging to miR-381 and miR-577, respectively (147, 148).

In addition to the signaling pathways mentioned above, there are some other signaling pathways associated with lncRNA in osteosarcoma. The NF-κB pathway plays a key role in cancer progression, which is regulated by a variety of mechanisms and controls cell growth, invasion and metastasis (149). The study found that the expression of NKILA, which reduced the proliferation, invasion and migration of osteosarcoma cells, was activated by the NF-κB pathway. NF-κB inhibitors can reverse the effect of knockdown of NKILA on cell migration and proliferation (150). In addition, targeting XIST can also inhibit cell proliferation and tumorigenesis by activating NF-kB and NF-kB-dependent PUMA signaling pathways (151). HIF-1α has been shown to regulate hypoxia gene expression through a signal transduction network. FOXD2-AS1 in the hypoxic tumor region can act as a miRNA sponge to regulate tumorigenesis. The expression of FOXD2-AS1 is up-regulated in osteosarcoma and is positively correlated with poor prognosis. HIF-1α can bind to the promoter region of FOXD2-AS, thereby increasing mRNA and protein levels (152). LINK-A may also act as an upstream activator of HIF1α and participate in the metastasis of osteosarcoma by up-regulating the HIF1α pathway (153).

## 6 Potential preventive and therapeutic effects of lncRNA in osteosarcoma

### 6.1 LncRNA in osteosarcoma diagnosis and prognosis

Although increasing numbers of current treatment alternatives are available for individuals with sarcomas, the efficacy of contemporary therapies has not changed over time. At present, the methods used to treat osteosarcoma include high-dose methotrexate, adriamycin and cisplatin before and after surgical resection. In addition, different combinations of etoposide, methotrexate, cisplatin, doxorubicin, and ifosfamide were also used to consolidate the treatment, but the results were not satisfactory (154, 155). Chemotherapy regimens consisting of ifosfamide and etoposide or gemcitabine and docetaxel have a certain effect on patients with unresectable recurrent diseases, but the overall event-free survival at 4 months is only 12% (156, 157). Adverse prognoses arise owing to the high risk of remote tumor dissemination and relapse. If malignancy were detected early and a precise prognosis was evident, there would be the potential for prompt targeted treatment and better overall survival statistics. Therefore, the presence of apposite clinical diagnostic or prognostic indicators would be extremely valuable. Numerous studies that have evaluated the role of lncRNAs in osteosarcoma have suggested that these molecules have promise for this application (**Tables 2**, **3**).

**Table 2:** Potential preventive and therapeutic effects of LncRNA in osteosarcoma.

LncRNA	Relevance	Role
MALAT1	Associated with a late pathological stage, remote tumor deposits and shortened overall survival	Acts as an autonomous prognostic factor for osteosarcoma
TUG1	Associated with adverse clinical prognosis	Acts as autonomous marker for overall survival and progression-free longevity
MEG3	Associated with individual survival	Acts as autonomous markers for a reduced overall survival time
FGFR3-AS1	Associated with elevated tumor dimensions, late Enneking stage and adverse clinical prognosis	Serves as a predict marker for metastasis potential and prognosis
HOTTIP	Associated with late-stage disease and the presence of remote tumor deposits	Acts as a potential prognostic marker and target for treatment in this clinical setting
FOXC2-AS1	Associated with adverse clinical prognosis	Acts as a possible treatment target
LUCAT1	Associated with drug resistance of chemotherapy drugs	Acts as a predictive biomarker in individuals with osteosarcoma
ODRUL	Associated with heightened osteosarcoma doxorubicin-resistance	Diminishes DXR susceptibility in osteosarcoma cells
LINC00161	Associated with apoptosis induced by cisplatin	Ameliorates the resistance of osteosarcoma cells
LINC00922	Associated with DXR resistance in osteosarcoma samples	The silence of LINC00922 inhibits osteosarcoma growth

**Table 3:** The way lncRNAs regulate drug resistance.

LncRNA	Ways to regulate drug resistance
FOXC2-AS1	Promotes ABCB1 expression, thereby increasing drug resistance in osteosarcoma
LUCAT1	Regulate drug resistance in osteosarcoma through the interaction between miR-200c and ABCB1
ODRUL	Diminishes DXR susceptibility in osteosarcoma cells owing to its ability to amplify ABCB1 expression
LINC00161	ameliorates the resistance of osteosarcoma cells by targeting the signaling axis comprising the miR-645-interferon-induced with tetratricopeptide repeats 2 (IFIT2)
SNHG12	Regulate drug resistance in osteosarcoma through its capacity to sponge miR-320a

Elevated MALAT1 in osteosarcoma tissue have been found to be associated with a late pathological stage, remote tumor deposits, and shortened overall survival, suggesting that this lncRNA could act as an autonomous prognostic factor for this tumor type (158). Significantly, MALAT1 levels within the serum could also be utilized in this regard. The degree of MALAT1 expression had a negative association with 5-year survival figures. In the cohorts with low and high expression, the 5-year survival statistics were 56.5% and 39.1%, respectively, a difference additionally shown with Kaplan-Meier survival curves (159).

An adverse clinical prognosis was robustly linked with amplified TUG1 expression, which was determined to be an autonomous marker of overall survival and progression-free longevity. When judged against preoperative values, serum TUG1 levels were noted to be markedly diminished in individuals following surgery, with the increase of TUG1 in serum identified in those patients exhibiting advancing malignancy or recurrence (39). Moreover, TUG1 knockdown in the osteosarcoma cell lines, U20S and Saos-2, induced by selective siRNA, leads to a declining cell replication and colony generation, heightened programmed cell death, and the arrest of the cell cycle at the G1/S phase. It is also associated with reduced POU class 2 homeobox 1 (POU2F1) expression, indicating its influence on the TUG1/mir-p-5p/POU2F1-axis in these cell types (160). When these data are combined, TUG1 could be used as a marker for prognosis, treatment, diagnosis, and monitoring purposes.

Overall survival was less in individuals with reduced, as opposed to amplified MEG3 expression (161). In patients presenting with osteosarcomas, low levels of MEG3 expression, a late pathological stage, and the presence of remote tumor deposits were all determined to be autonomous markers of reduced overall survival time.

Elevated tumor dimensions, late Enneking stage, and adverse clinical prognosis have been linked to the amplified expression of FGFR3-AS1 (136). When identified within cells, FGFR3-AS1 promotes amplified FGFR3 expression through a mechanism involving the coupling of antisense with the 3’UTR section of FGFR3. Additionally, when the FGFR3-AS1 shRNA expression plasmid pGPU6/GFP/Neo is used to knockdown FDFR3-ASi in MG63 cell lines *in vitro*, advancement of the cell cycle and cell replication are inhibited (136). Correspondingly, prognosis may be predicted using FGFR3-AS1 in the clinical context of osteosarcoma.

The late-stage disease and the presence of remote tumor deposits have been linked with the amplified expression of HOTTIP in samples from osteosarcomas. Increased expression of HOTTIP has also been related to high mortality and is an autonomous prognostic indicator for overall survival in individuals with this tumor type. Hence, this lncRNA is a potential prognostic marker and target for treatment in this clinical setting (162).

Amplified MIR100HG, HOXD-AS1, EWSAT1, and LMCD1-AS1 expression, in addition to several further lncRNAs, have been determined by Kaplan-Meier curves to forecast adverse clinical outcomes in individuals with osteosarcoma (163, 164). Statistical analyses, such as univariate and multivariate Cox regression, have also indicated the prognostic value of lncRNAs, including LOXL1-AS1, EWSAT1, ILF3-AS1, CBR3-AS1, DLX6-AS1, UCA1, DICER1-AS1, and Ftx in this form of malignancy (164).

### 6.2 LncRNAs in osteosarcoma chemotherapy and chemotherapeutic drug resistance

The principal types of therapy applied to malignancy include operative resection, and treatment with either chemotherapy or radiation. However, many osteosarcomas fail to demonstrate significant regression following conventional chemotherapy or radiotherapy regimens and may develop resistance to therapies (165). Resistance to chemotherapeutic agents can be categorized as either acquired or primary (166). The latter is already present, whereas the former arises during treatment following the adaptation of malignant cells (167). This occurs via a complicated mechanism to which numerous factors contribute, together with impaired processes of programmed cell death and autophagy (168). In osteosarcoma, the lack of efficacy of chemotherapeutic endeavors predominantly arises owing to secondary resistance to drugs such as doxorubicin (DXR) and cisplatin (169). In some cases, using lncRNAs as targets for therapy has been shown to attenuate the issues of pharmaceutical resistance and enhance the susceptibility of the tumor to treatment (170) (**Table 2**).

An abundance of research has concentrated on utilizing genetic and molecular analytical techniques to investigate the mechanisms underpinning chemoresistance in osteosarcoma. The factors identified factors include a spectrum of intrinsic biological changes, including the heightened expression of members of the ATP-binding cassette (ABC) membrane transporter family, aberrant metabolic pathways, disruption to the governance of the cell cycle, and abnormal apoptotic processes (171, 172). These alterations can inactivate pharmaceutical agents, diminish the accrual of drugs within the cells, induce chemoresistance via the effects on malignant stem cells, and cause dysfunctional signal transduction pathways, the aberrant control of miRNAs and drug resistance due to aberrant cell death and autophagy (173, 174). The ABC transporter protein family members rely on ATP for the efflux of pharmaceutical agents (175). Their expression is influenced by lncRNAs, which consequently affect the resistance of malignant cells (176, 177).

In samples from human osteosarcomas and in the cell lines MG63 and KH-OS, which exhibit DXR resistance, there is excessive expression of long non-coding RNA Forkhead box protein C2 antisense 1 (FOXC2-AS1) (169). In human tissue samples from osteosarcomas, this finding is associated with an adverse clinical prognosis, and *in vitro*, it is linked with the enhancement of resistance to DXR in the same cell lines. Conversely, FOXC2-AS1 knockdown promotes the susceptibility of cells from osteosarcomas to DXR and reduces osteosarcoma cell resistance to DXR in cell lines, including MG63/DXR and KH-OS/DXR, through FOXC2 inhibition. ABCB1 expression is also promoted by FOXC2, which adds to the drug resistance seen in osteosarcomas (169). Thus, the amplification of ABCB1 expression is a common pathway to DXR resistance in cells from osteosarcomas caused by FOXC2-AS1 and FOXC2. FOXC2-AS1 appears to have two potential roles. Firstly, it may be a useful marker in patients with osteosarcoma to highlight potential DXR resistance, and secondly, it represents a possible treatment target for increasing tumor susceptibility to DXR.

Cell lines from osteosarcomas that display resistance to methotrexate, an extremely potent chemotherapeutic agent used in this type of neoplasia, have been demonstrated to contain amplified lung cancer-associated transcript 1 (LUCAT1) expression (178). In addition, LUCAT1 can interact with ABCB1 through miR-200c, which combines with the 3’UTR of ABCB1 and is regulated by LUCAT1. Accordingly, if elevated levels of LUCAT1 were detected in human osteosarcomas, it can be used as a predictive biomarker in individuals with osteosarcomas.

Patients with osteosarcomas and tumor dissemination to the lungs and a poor response to chemotherapy demonstrated heightened osteosarcoma doxorubicin-resistance related to up-regulated lncRNA (ODRUL) expression. Notably, the lncRNA ODRUL may diminish DXR susceptibility in osteosarcoma cells owing to its ability to amplify ABCB1 expression (179). Furthermore, several lncRNAs, such as ENST00000563280 and NR-036444, were recognized from a lncRNA-mRNA co-expression network. These engage with genes including ABCB1, HIF1A and FOXC2, which may contribute to elevated levels of osteosarcoma resistance to DXR (180).

Sustained cell survival necessitates prompt and apposite reparation of any injury to DNA. Many miRNAs might control the responsiveness of osteosarcoma cells to treatment with radiation by influencing these mechanisms. Apoptosis brought about by cisplatin is significantly influenced by lncRNA, long intergenic non-coding RNA 161 (LINC00161). Additionally, this lncRNA ameliorates the resistance of osteosarcoma cells by targeting the signaling axis comprising the miR-645-interferon-induced with tetratricopeptide repeats 2 (IFIT2) (181).

DXR resistance in osteosarcoma samples has been linked to amplified LINC00922 expression, the silence of which inhibits osteosarcoma growth. One of LINC00922’s functionalities is to sponge miR-424-5p, which decreases transcription factor TFAP2C expression. Consequently, it has been proposed that a feedback loop, including TFAP2C, LINC00922, and miR-424-5p, is important in DXR resistance (182).

CTA levels have been demonstrated to be diminished in cells that are resistant to DXR (130). Significantly, enhancing CTA levels led to a notable rise in two recognized miR-2010 targets, including caspase-8-associated protein 2 and apoptosis-inducing factor, mitochondrion-associated 3 (183). Correspondingly, the reparation of CTA may therefore potentially diminish cell survival through competitive bonding with miR-201, enhancing apoptosis and suppressing autophagy. Moreover, increased SNHG12 transcript levels have been noted in cells from osteosarcomas that demonstrated DXR resistance, as opposed to those that are susceptible to this drug at cytotoxic levels. The mechanism of action of SNHG12 in cells from osteosarcomas arises through its capacity to sponge miR-320a, which has an inhibitory influence on the expression of MCL1 (101).

Anomalies in drug breakdown form a second key pathway that brings about resistance to pharmaceutical reagent. Important enzymes within these metabolic pathways can be affected by several lncRNAs. Enzyme systems that are key to the activation and inactivation of many drugs include the cytochrome P450 (CYP) system, and the glutathione-S-transferase (GST) and the uridine diphosphoglucuronosyltransferase (UGT) superfamilies (184). Numerous pharmaceutical reagents used in the treatment of malignancy have to be activated by the body’s metabolism, which could lead to drug resistance if this process is impaired by neoplastic cells (167). Elevated CYP3A4/5 levels have been associated with adverse clinical outcomes in individuals with osteosarcomas (185). Furthermore, after the administration of DXR or cisplatin, glutathione-S-transferase P1 (GSTP1) levels were found to be elevated, and amplified GSTP1 expression in SAOS-2 cell lines from osteosarcomas was linked with heightened vulnerability to resistance to these two pharmaceutical reagents (186).

## 7 Summary and future prospects

Osteosarcoma, an extremely aggressive malignancy, is typically characterized by invasion at the site of origin, remote dissemination, and recurrence. There is an exigent requirement to comprehend the disease processes underlying this cancer type to facilitate the innovation of *de novo* clinical treatments. The above review has detailed the abundance of evidence that substantiates the possibility that lncRNAs are key actors in the onset, growth, invasion, systemic dissemination, and resistance to pharmaceutical agents exhibited by osteosarcomas. The expression amplification or downregulation of these lncRNAs means that they can display either oncogenic or tumor-suppressive properties, respectively. Examples include HOTAIR, MALA T1, H19, TUG1, MEG3, and TUSC7.

A range of pathways have been identified concerning lncRNAs, such as host gene targeting, signal pathway involvement, and ceRNA activity. Several lncRNAs have been determined to function as autonomous indicators of prognosis, including the likelihood of the presence of drug resistance to modern treatments such as DXR and cisplatin. Such data pertaining to lncRNAs are encouraging for the future innovation of clinically applicable biological indicators for the diagnosis and prediction of survival, as well as potential targets for treatment. LncRNA can be targeted by using small interfering RNA, antisense oligonucleotide, ribozyme, aptamer, and miRNA. These methods have long been evaluated for targeting key cancer-related genes, and they are at different stages of clinical trials. For example, flavonoids are negatively correlated with the risk of colorectal cancer. These bioactive compounds can target the lncRNA/Wnt pathway to reduce the side effects of anticancer drugs (187). Simvastatin, a potential therapeutic drug for colorectal cancer immunotherapy, promotes anti-tumor immunity by inhibiting lncRNA SNHG29-mediated YAP activation and inhibiting PD-L1 expression (188). In addition, due to the ability of oligonucleotides to enter cells and specifically target RNA that cannot be accessed by antibodies, oligonucleotide drugs show stronger target specificity than small molecule drugs and reduce potential side effects. Therefore, lncRNA-based oligonucleotide drugs are being studied and are expected to make further progress. Nevertheless, research investigating the role played by lncRNAs in osteosarcoma is still in its infancy and predominantly in the pre-clinical phase. Despite all this, recent experimental data are extremely promising regarding the utility of lncRNAs as future diagnostic and prognostic clinical indicators, and as treatment targets in patients with osteosarcoma.

## Author contributions

SH: Writing – original draft, Writing – review & editing. XH: Writing – original draft, Writing – review & editing. GL: Supervision, Writing – review & editing. SW: Funding acquisition, Writing – review & editing.
